# Abstracts &c.

**Published:** 1889-07

**Authors:** 


### ABSTRACTS AND GLEANINGS.

**On the Use of Hypnotics, Sedatives, and Motor Depressants in the Treatment of Mental Diseases.**—The author deduces the following principles of general application:

1. Make up your mind clearly from the symptoms present whether your patient needs a pure hypnotic, a general nervous sedative, or a simple motor depressent before you use any of these drugs.

2. Use all such drugs experimentally in each case at first, and watch their effects not only on the higher nervous functions, but on all the organs and their functions, and on the general organism.

3. Even when there is sleep and quiet produced for the time with no apparently bad results, look to the general feeling of *bien-etre*, the recuperative energy, the expression of face and eyes after their use, and see if there is any undue reaction, as if some energy that must have an "outlet" were merely being "suppressed" for the time being.

4. Stop using such drugs as soon as possible, trying experimentally how the patient gets on without them.

5. Keep asking in every case: "Are we sacrificing in any degree the hlghest functions of mental inhibition by their use?"

6. Never omit general measures for the restoration of the health, nutrition and higher nervous functions while you use such remedies.

7. Paraldehyde is the purest and least harmful hypnotic yet introduced when the insomnia is marked and intractable. Urethan and sulphonal cannot compare with it. Opium and chloral have special dangers and disadvantages.

8. Use the bromides as accentuators and prolongers of the effects of other drugs, and in order to be able to employ smaller doses than otherwise.

9. A combination of cannabis Indica and the bromides is the best and least harmful of the general sedatives.

10. Hyoscine is the best pure motor depressant, but it needs great care.

11. We never should narcotize an insane patient or one threatened with mental disease.

12. It is as dangerous to use mere anodynes by the mouth or subcutaneously to relieve mental pain, as to subdue bodily pain by these means only, perhaps more so.

13. It is generally far better therapeutics to enable your patient to bear his mental pain and the effects of his insomnia by improving his general nervous tone and the nutrition of his body, than merely to produce quiet and sleep by drugs.

14. It is commonly a safer thing for the patient, and tends more towards natural recovery from his disease, to provide a physiological outlet for morbid motor energy, than merely to depress it directly by drugs.

15. It is almost always preferable to treat critical exhaustion, irritability and undue reflex excitability by rest and by improving the fattening and nutrition of the body than by continuous sedatives, the great exceptions being the treatment of epilepsy and convulsive affections by the bromides.—*Amer. Jour. Med. Science, April, 1889.*

---

**A Growing Science.**—"It seems to me," muses one of the characters in "*Robert Elsmere*," that in my youth people talked about Ruskin; now they talk about drains." This strikes the keynote of public opinion as we find it at the present time. The man of to-day no longer gluts his appetite with superstitious ideas, destroying his taste for facts, but instead is met on every side with a vast array of statistics, which are more than convincing—which are startling; and he is told in the plainest possible terms, that if he would prolong his own life and preserve that of his children, certain immutable laws of hygiene and sanitation must be observed.

No science has wrought so many changes or grown so rapidly during the past twenty years as that of sanitation, nor has any other been so constantly dinned into the mind of the people at large. Many of its laws have been known for centuries, but it is only during recent years that they have been intelligently understood in their true meaning.

It was difficult to say exactly along what lines this progress has occurred, for like all great movements it is the resultant of many and varied causes.

The germ theory of disease, the supposition that minute organisms may enter the blood and produce sickness and death at first set many to thinking, and better still, has more recently set many great minds *to working*, and we know as a positive fact that there are micro-organisms invariably found in certain diseases which are peculiar to them, and which will produce them on reinoculation. To destroy these germs in the human body has not yet been found an easy task, but the fact that certain substances when brought in contact with them outside of the human body will destroy, or at any rate hinder their growth, has opened up a field of prevention, the limit of which has not yet been arrived at. Antiseptics—poison destroyers—for use in and out of the human system, are at a premium in the exchanges of science, and it were not too much to say that we are on the eve of the discovery of valuable specifics for some of our commoner epidemic diseases.

Taking advantage of the crude facts already ascertained we have been able to apply with gratifying success the more powerful antiseptic substances to prevent the recurrence of disease in certain infected localities.

Antisepsis is the scientific term for *cleanliness*—a word much better understood by the masses, who will aid their physicians in their humane endeavors only in so far as they comprehend their meaning. Hence the necesity for, and hence the valuable services performed by the health primmer in our public schools—a book always interesting to the child, and, in an era of common sense, to be considered far more valuable than much that has hitherto been used to expand the youthful mind. Hence the necessity for such teaching in the university, and for a special chair in medical colleges; for though physicians should be natural sanitarians they should not be expected to become the tutors of their private patients, who will not care, as a rule, to send a second time for the only man who ever told them that their house was dirty and drinking-water impure.

Organization in health matters is the safeguard of a community, and we find that this is brought about not so much by medical men, who have preached ineffectually for so many years, but by merchants and business men generally, who now look to some other cause than Providence when they find that their pockets as well as their lives are threatened by the periodical visitation of some dreaded scourge. Health boards are being demanded and created for States and cities, and conferences are called to determine plans of concerted sanitation. No State in which disease may become epidemic can afford to face the civilized world and assert that she has no organized health authorities.

These authorities, it is true, often differ in their views as to what sanitation is, and how disease is to be prevented, but as there is an increased tendency in all to follow those methods of prevention laid down by close scientific investigators, such as by fumigation, disinfection, and, the greatest of all, cleanliness, we may entertain the most sanguine hopes for the future of preventive medicine.—*New Orleans Medical Journal.*

The *London Lancet* says that a small light attached to the end of a glass rod is reflected equally through the entire rod to the other end, illuminating the interior of the larynx sufficiently for laryngoscopy. If this luminous glass rod is applied to the sclerotic, the interior of the ball can be examined in the same way as by using an ophthalmoscope, the structure of the posterior part of the vitreous body being very well seen and studied. As the glass rod remains cold, it can be employed in operative surgery to light the natural and artificial cavities.

**Cocaine in the Extraction of Teeth.**—A physician in this city prepared himself to suffer no pain in the extraction of his teeth by injecting, by use of the hypodermic syringe, a four per cent. solution of cocaine in the gums, and taking large draughts of whisky. He suffered no pain, but described the sensation as one of constant percussion, attended by a pressure even after the teeth were extracted. During the operation he retained consciousness, and immediately afterwards was competent to resume his business duties.

A much better method, however, is the use of the preparation suggested by Dr. G. S. Staples, in the *Archeves of Dentistry*. Dissolve twenty grains of cocaine in one ounce of ether, and add one ounce of oil of peppermint; by the local application the good results are obtained in a short interval.—*Indiana Med. Journal.*

**Prolapse of the uterus—Hysterroraphy.**—Case 1. Mrs. A., aged 60, Irish, had prolapsed uterus after the birth of her first child. From this she experienced a great deal of suffering which was increased by a ventral hernia, which appeared twenty years ago. The uterus has been greatly enlarged within the past two months, and it was found impossible to replace it.

On examination it was discovered to be prolapsed, the cervix dry and fissured bi-laterally, and a large circular erosion on the anterior surface. The fundus was large and congested.

LeFort's operation was performed, the anterior and posterior walls of the vagina were united in the median line by catgut sutures above and silk below, thus leaving a small canal on either side. The perineum was then united by wire sutures, according to Emmet's method. A dressing of iodoform and bichloride gauze was then applied. During the twenty-four hours following the operation the patient was kept on fluid diet, and the bowels were moved on the evening of the second day. The wound was daily sprayed with solution of bichloride, 1 to 5,000, and the dressings reapplied. The stitches were removed from the perineum on the fifth day, when the wound was found perfectly healed. A vaginal douche of two quarts of sublimate solution, 1 to 1,000, was then given twice daily. The vaginal sutures were removed May 3, and complete union of the parts was found.

Cass 2. A. M., aged 25, domestic, had a double ovariotomy performed on Feb. 13. She still experienced constant headache, and continued to menstruate, which latter condition was supposed to be due to the persistent retroflexion. Hysterroraphy was performed April 26; an incision, 2½ inches in length, was made in the median line between the umbilicus and pubes, the fascia and peritoneum were incised separately, and a sound was kept in the bladder, as adhesions were known to exist between that organ and the uterus. These adhesions were broken up, and the uterus was found to be retroverted. A double twisted silk suture was then carried through the integument, fascia, peritoneum and base of the round ligament on one side, across the fundus of the uterus, and through the same structures on the opposite side. The wound was then closed with three wire and five catgut sutures. The patient was put on a liquid diet and the pain relieved by an ice coil applied over the abdomen.

On May 19 the patient was able to sit up for the first time since the operation and was feeling well.—Dr. Cushier in *International Journal of Surgery.*

**Ammonium Chloride for Quick Relief from Drunkenness.**—It is claimed (*Albany Med. Annals*, March, 1889) that half a teaspoonful of chloride of ammonium in a goblet of water will almost immediately restore the faculties and powers of locomotion to a man who is helplessly intoxicated. [We know this treatment is good. The remembrance of it may be of service to some subscribers who are getting ready to attend the sessions of their State Societies. We have several times had occasion to prescribe it on such occasions.—ED.]—*Va. Med. Month.*

**Infection of an Infant through the Milk of a Tuberculous Nurse.**—Dr. Steigenberger, of Buda-Pesth, has recorded a case of tubercular infection through the nurse's milk in the *Pesther medicinisch-chirurgische Presse.* The facts of this intereting case are summarized as follows: An infant, aged five months, of healthy parentage, developed caseating cervical, glandular abscesses, of a distinctly tubercular kind. Microscopical examination verified the microscopical diagnosis. Inquiry elicited the fact that the infant had been nursed for a period of four weeks by a woman who had to be discharged on account of phthisis, with abundant expectoration. The etiological relationship was thus clearly established.

The infection of human beings through the milk of tuberculous animals has been repeatedly shown, and there is, of course, no reason why the human milk should not carry with it the same pathogenic power. But, so far as we are aware, the above case is the first instance in which this method of transmission has been actually observed to occur. The inference is obvious, namely, to exercise the greatest possible amount of care in the selection of wet-nurses.—*New York Medical Record.*

In view of the liability to the formation of a furuncle after the removal of a plug of of cerumen from the ear by simple syringing, Lowenberg (*Gaz. hebd. des Sci. Med.*) recommends that the mass be treated previously for a day or two by instillations of an antiseptic solution made after the following formula:

℞ Boric acid, 7 parts; Glycerin; Distilled water, ää. 100 parts. M. This solution should be warmed and dropped into the ear from a test tube. It is to be applied twice a day, and the liquid allowed to remain in the ear for fifteen minutes. It will increase the deafness for a time, on account of an augmentation of the plug by imbibition, but it softens the mas and facilitates its expulsion.—*Ib.*

**Ignipuncture of the Tonsils** is practiced by Dr. Wilhelm Roth, of Fluntern, (*London Lancet*) in order to reduce their size without risk of troublesome hemorrhage, which is not uncommon, especially in young subjects.

This has also been recommended by Krishaber and by Verneuil. The tonsils and neighboring parts are first brushed over with a ten or twenty per cent. solution of cocaine. The finest point of the thermo-cautery, heated to redness, is then inserted to a depth of about five millimeters in three or four spots a few millimeters apart from one another on the tonsils. The instrument is not allowed to remain more than one or two seconds in the tissue.

The whole operation, including both tonsils, can be performed in a very few minutes without any bleeding, and with scarcely any pain. It must be repeated four or five times at intervals of two or three days, and this is usually sufficient to cause the tonsils to return to their ordinary condition.—*Ib.*

**A new Objection to the use of Chloroform for Anæsthesia.**—Americans have steadily kept their allegiance to ether as an anæsthetic in surgical procedures, for various and good reasons. Now comes from Germany a discovery that must needs confirm their belief in the advantages of this agent. Dr. Zweifel, in a late issue of the *Berliner Klinische Wochenschrift*, calls attention to the danger which exists in the administration of chloroform by gas or lamp-light. It appears that the vapor is decomposed, giving rise to an irrespirable and irritating gas which promotes respiratory lesions which dangerously complicate the operation. Of nine laparotomies performed under the conditions already mentioned, only two remained free from bronchial and pulmonary troubles, such as bronchitis, tracheitis, and catarrhal pneumonia. In one case the fatal termination could be directly ascribed to a pneumonia produced in this manner. As soon as the use of chloroform was abandoned, and ether substituted for it, these complications ceased to occur, but were renewed in one case in which chloroform was administered by mistake. Hence the author pleads for ether anæsthesia in such cases as must undergo operation by gas or lamp-light. He is in the habit of inducing anæsthesia in the sick room with a mixture of chloroform, ether, and alcohol, and continues the anæsthesia during the operation with ether alone, thus avoiding completely the dangers of chloroform vapor.—*Int. J. Surg.*

**The New Antipyretic, "Pyrodin"—A Warning.**—Under this name a new drug has been introduced, which has undoubted temperature-reducing properties of a high order, the practical application of which, however, is much interfered with by its toxic action. Pyrodin contains as its active agent acetylphenylhydrazin ($C_6H_5N_2H_1C_9H_3O$) a crystalline powder very sparingly soluble in water. According to the clinical and experimental observation of Dr. Dreschfield, of Manchester, which have been confirmed by M. Lepine, of Lyons, (as stated by the *British Medical Journal*), pyrodin acts in the same manner as, but more powerfully than antipyrin, antifebrin and phenacetin; and has also been used effectively in migraine and other forms of neuralgia, as in the lancinating pain occurring in locomotor ataxy (Lepine), Great caution, however, is required in its administration, as it is apt to produce jaundice, followed by anæmia, and even more serious symptoms due to hæmoglobinæmia. Milder toxic symptoms have occasionally followed the administration of acetanilid or antifebrin, and also of phenacetin; but, as phenylhadrazin is a much more powerful poison than anilin, so also, are the toxic properties of its acetyl compound much greater than those of acetanilid. In face of the poisonous qualities of pyrodin, we must warn the profession against the use of this drug generally. In exceptional cases, and where other antipyretics have failed, it may be useful, but great caution should be used. Small doses only should be given, and at sufficiently long intervals to enable one to watch any toxic effects, with the first appearances of which the drug should be stopped.—*Ib.*

**Tænia.**—The treatment of tænia in children is not easy. I have constantly succeeded by making them pursue the following treatment: Take for example, the case of a child five years old:

1. Abstinence from supper the evening before.
2. The following morning this preparation:

℞ Ethereal extract of male fern, 4 grammes.
Calomel, 40 centigrammes.
Sugar, 8 grammes.
Gelatin, q. s.

Make into a jelly of ordinary consistency. Children take this kind of electuary very readily.

It has been recommended that the patient have a lavement of salt and water, or a purgative lavement, as the worm is passing the anus. This s easy in theory, and dificult in practice. In the case of one patient the worm issued at 1:30 p. m., and though six lavements of salt and water were given, and 60 grammes (2 ounces) of mercurial honey, no result was obtained till 5:40 p. m. It is easy to understand, in fact, how difficult it is for patients to take lavements in the sitting posture; since it is necessary that they shall be over a vessel of warm water to prevent the worm breaking by its weight, and since, when the worm is at the anal orifice, it is almost impossible to keep up the lavement in order that this may provoke intestinal contraction. Whether you employ this or that tanifuge, even if you call into use the whole therapeutic arsenal, I am persuaded that the chief causes of failure are due to the too tardy administration of the purgative after giving the athelmintic which is designed to stupify or destroy the worm; and as evidence of this, I may allude to the almost constant success which I have had with the tænifuge capsules in which the calomel is administered along with the male-fern.

I do not know that we have as yet found the ideal tænifuge, and I hope that pharmacists will continue their endeavors to extract from the known active anthelmintics, alkaloids which, in small volume, will produce without danger or harm, the expulsion of the tapeworm.—*Dr. Duchesne in Medical Age.*

[We have found no remedy so efficient as *Kooso*, whether in adult or children. The dose for an adult is a half-ounce on an empty stomach at morning after a purgative, no supper being taken the previous evening. The kooso must be pulverized and in sealed bottles, as the strength will not keep in open drawers. To children and weak stomachs should be taken in broken doses every hour. ED. OF RECORD.]

Dr. Stewart (*Peoria Med. Mo.*) states that, for the relief of facial neuralgia, ergot injected hypodermically is incomparably superior to either aconite or gelsemium. Generally one injection relieves the pain permanently. He puts it into the temple, as nearly over the seat of pain as convenient. He uses the plain extract, which he has made so that one minim represents two grains of ergot, throwing in eight to twelve minims, blood-warm, at one injection, without diluting. The extract must be pure and reasonably fresh. If old, it is inefficient and irritating. Injection of the fresh product causes more or less pain for a few minutes, but when it disappears the neuralgia has usually disappeared, not to return.—*Pop. Science News.*

**Electricity as a Cure for Cancer.**—Surely, of the wonderful effects ascribed to electricity, there seems to be no end. Dr. J. Inglis Parsons of London thinks that he has found in that agency a means of rendering cancer inert. His idea is that cancer cells, although they grow like noxious weeds and choke the normal elements are really of a lower vitality than the latter, and hence are susceptible of destruction by electrical currents that the healthy cells are able to withstand. He has tested his theory in four cases, which he reports in the British Medical Journal for April 27th. The patient is anæsthetized, and the current is passed through the tumor and all the tissues for some inches around it by means of fine insulated needles, so as not to injure the skin. A battery of seventy cells is used, with an electro-motive force of 105 volts. To begin with, a current of an intensity of 10 milliamperes is employed, and this is gradually increased to 600 milliamperes, being flashed through the growth in every direction from 50 to 100 times, according to circumstances. In only one case was it considered unsafe to use a current of more than 250 milliamperes, and that was one of mammary cancer in a woman sixty-three years old, who had a presystolic bruit and a weak, intermittent pulse, and even she was able to endure a current of 600 milliamperes passed through a secondary growth in the axilla. It is not so much to the strength of the current, although that seems to be essential, as to its sudden interruptions that the destructive effect on the cancer cells is attributed. The tumor does not disappear altogether, but remains as an inert mass; at least, in the cases reported, the growths remained quiescent for periods from three and a half months to six months. In case of recurrence, the author states, he would not hesitate to use a current of still greater intensity. It is said that the patient is usually able to go about his customary employment on the day following the operation.—*N. Y. Med. Jour.*

**Salts of Phosphorus.**—Dr. Joseph Eichberg, President of the Cincinnati Medical Society, read a paper recently on the acid salts of phosphorus, which was intended to call special attention to their value in disorders of digestion. The value of the element phosphorus in the various compounds were referred to; its physiological uses in alkaline, acid and earthy combinations were illustrated; and a comparison was instituted between the nitrogen and phosphorus radicles, as shown by the varying affinity for oxygen. It was stated that this fact alone was proof of the important role played by any chemical in the tissue changes of the body, the apparent readiness of change from one form of radicle to another being observed only in those elements whose functional activity was great. In regard to the form of administration of phosphorus, attention was called to the fact that this varied with the nature of the disease. The hypophosphites in phthisis and wasting diseases, the phosphate of zinc and phosphorus itself in emulsion for nervous diseases, and the acid salts of phosphorus for digestive disturbance, were the forms in which the greatest benefit would be derived from the use of the remedy. Phosphoric acid and its compounds possessed an advantage over the other mineral acids in that, even in a concentrated form, they were essentially non-corrosive. Considerable benefit had been derived from a compound called "crystalized phosphates."—*N. Y. Med. Jour.*

**The Treatment of Croup.**—Dr. J. B. Johnson, Washington, D. C., writes in *Southern Clinic*:

The following formula has been a standard prescription of mine for croup for several years. It relieves all the symptoms of the disease with greater promptness and certainty than any other mixture I have ever used. I give it in teaspoonful doses to infants six or eight months old; and to those of three or five years old I give dessertspoonful doses every ten or fifteen minutes, until free emesis is produced; and then continue it at longer intervals until a cure is established. The formula is as follows:

| | | |
|---|---|---|
| ℞ | Muc. gum acacia | ℥ ij. |
| | Balsam copaiba | ʒ j. |
| | Ext. ipecac, fl'd | ʒ j. |
| | Potassii iodidi | ʒ j. |
| | Pulv. potassii chlorati | ℥ j. |
| | Syrup simplex | ℥ ss. |
| | Aqua distil. | ℥ j. |

M. S. Shake well. Dose, a teaspoonful every ten or fifteen minutes until free vomiting ensues, ; and then continue the same dose at intervals of a half-hour or hour, until the disease yields.

**Cured by Thermometer.**—The following, from *Scribner's Magazine*, is a pendant to the well-known story of Sir Humphrey Davy and the paralytic patient; the importance attached to a clinical thermometer by those in ignorance of its office amounts to a superstition. They close their lips tightly upon it. Their eyes roll wildly about the room. They believe that the tube contains some mighty gas or metal of mysterious power. "There aint much taste to it, docther," said one of these credulous fellows, "but I s'pose its terrible strohng." Dr.——, who is something of a wag, encouraged the man's faith in the occult virtues of the thing, and with remarkable results. After the first "dose," the fever abated. The treatment was continued, and the patient actually recovered, cured by thermometer, administered *ter in die*, without further drugging.—*Med. Times.*

**A Remedy for Neuralgia.**—It is claimed that a few drops of the following: Eau de Cologne, ether, chloroform, ää ʒ iij, poured on a handkerchief previously wetted with cold water, and placed on the seat of a neuralgic pain, give instantaneous relief. It is also very efficacious for nervous headache. A burning sensation is felt at first, but quickly disappears.—*Ex.*

**Notes on the use of the Salicylates.**—In common with other observers I have been impressed with the great efficacy and widespread applicability of Salicylic Acid and its compounds. Some of their therapeutic indications have already been told, others have received little if any attention. These I desire to briefly to comment upon.

In trifacial neuralgia, particularly, the Salicylate of Soda has given me more satisfactory results than any one remedy. The adult dose is 15 or twenty grains every hour or every two hours until relief is experienced. It is seldom necessary to repeat the larger quantity more than two or three times, while the results seem to be uninfluenced by the etiology of the disease, provided, of course, no pressure exists either at the origin or along the track of the nerve. Although lumbago cannot be properly classed with neuralgic affections it may be stated that for this it is the first remedy to be employed.

This drug has lately acquired some little reputation from its power to relieve headache, for it has here a very general application and is to be given in the same doses. Many of the intracranial varieties are of course beyond its reach, yet that variety known as migraine is very often relieved, or in fact, absorbed by one or two large doses of the salt, preceded by a small hypodermic of morphia. It undoubtedly relieves many of the gastric forms of headache through its anti-fermentative properties.

The quality which this drug possesses of allaying decomposition and allied processes is quite apparent to those who have employed it for such purposes. In cases of simple overloaded stomach, as well as in certain forms of dyspepsia, a few doses of the soda salt often prove sufficient. The same applies to intestinal indigestion, though here we have found the salicylate of phenol (sàlol) of more general utility.

In all affections, whether acute or chronic, the indications are: To first remove from the gastro-intestinal tract, preferably by mercurial and saline, all foreign material and then to keep the drugs in constant contact with the mucous membrane by means of frequently repeated doses. For such troubles the other and newer antiseptics seem to possess little, if any superiority over the salicylates.

In the severe forms of what are known as "colds" where there are chills, severe headache, muscular soreness and pain, an elevated temperature and perhaps an oppressive bronchitis, the relief experienced from the soda salt is so great that its use has now become with me a matter of routine. It is invariably given and is sure to afford relief. The average dose for adults is 20 grains every two hours until the symptoms abate.

During the past year or two I have men with certain painful forms of tonsillitis, which, from the histories obtained, appeared to be of a rheumatic nature. These succumb readily to similar treatment and without any local medication whatever.

Although the salicylates have been known to call forth a skin eruption, I have, nevertheless, employed them in various cutaneous affections which possessed no rheumatic element. They are certainly remedial in urticaria, while in herpes zoster and some forms of eczema they are worthy of trial.

It is a well established fact that many diseases are produced or intensified by faulty elimination of partly converted or waste products. As the salicylates arrest decomposition, so also do they assist in the elimination of toxic compounds. Notably is this the case in lithæmic conditions. They seem to have a direct solvent effect upon urea and uric acid circulating in the blood, without injury to its normal ingredients. Its indications in this connection are many. In these affections salicylic acid is rendered more effective and acceptable by combination with the salts of potash in sufficient quantity to form a neutral or alkaline solution as the indications demand.

It is not in my opinion an antipyretic in the true sense of the word. That it reduces temperature in some cases is certain, but I believe it does so, not by reason of its power to lower arterial tension, or some effect which it may have upon the nerve centres, but by its action upon the toxic ingredients of the blood. For this reason it is of decided value in a great variety of septicæmic conditions.

As a rule, no untoward effects have resulted from the use of the various forms of the drug, though occasionally in cases requiring a few large doses there has been some cardiac depression. This may be prevented by the simultaneous administration of ammonia or some form of alcohol. In most asthenic conditions and in the later stages of fevers it is not usually indicated, while in children it is somewhat less effective than in adults. I have made no reference to its use in rheumatic conditions believing them to be already understood and appreciated, yet I will venture to state that I believe the bad effects of the salicylates upon the heart to have been unduly exaggerated. I trust that in the affections above noted the experience of others will be found to coincide with my own.—Dr. J. J. Berry in *N. E. Medical Monthly*.

**Hypertrophy of the Tonsils and its Treatment.**—Chronic tonsilitis, or hypertrophy of the tonsils, proceeds from two causes. A large number of cases are the result of a low form of inflammation occurring in childhood. The structure in childhood is very prone to become inflamed. If the tonsils are considerably enlarged, it is important to remove a portion of each. You should never speak of "cutting out the tonsils," as this sounds very alarming to the patient and his friends. Say that you mean to remove only "the diseased and enlarged portion." It is a consideration when you should do this. How much enlargement should there be before the operation is performed? First of all, the question of size is entirely relative. In a large throat the tonsils may grow to a considerable size, and the patient still do quite well. In a smaller throat this would not likely be the case. If the tonsils touch each other, you can have no doubt as to the propriety of taking away a piece. If adult patients come to you with the tonsils slightly enlarged, it is an important question whether you should cut off a portion or not. If the enlargement is associated with frequent attacks of acute inflammation, you ought then to cut away a piece. There is another condition which requires a similar proceeding. When the follicles of the tonsils are much enlarged, you cannot cure it except by taking off a section, which may not be more than one-eighth of an inch thick. You thus clear away the walls of the deep follicles and get a flat instead of a "worm-eaten" surface. As to the method of operating, many surgeons do it with a bistoury, and Sir William Ferguson, a great surgeon, for whom I had the greatest admiration, used to perform it in this way, but it was terrible to see the patient struggling with a mouth half full of blood before the operation was completed. Great surgeons will do all they can with a knife instead of what they call a "machine." I always perform the operation, however, with "a machine"—a tonsillotome. The particular form I use is a modification of Physick's. The great advantage of this is, that its mechanism is quite simple, and my modification enables the handle to be fixed on either side of the blade, so that the operation may always be performed with the right hand if the operator desires. As a general rule, lightness of touch is the chief desideratum in operating, but in tonsillotomy it is the reverse. Heaviness of touch is the important thing. The tonsillotome must be pressed well over the tonsil, which is also to be projected into it by pressure with the left thumb placed under the angle of the jaw. I once had a colleague who could do very little else, but he took off tonsils marvellously, and as I watched him I observed that it was this heaviness of touch that made him so successful. If you don't attend to this you will not take off nearly so much as you desire. Patients have come to me a week or a fortnight after the performance of the operation by another surgeon, saying that the tonsil had been removed but has grown again! This, of course, means that enough was not removed at the operation. It is most important to take off enough. Hemorrhage from this operation is rare, but it has occurred, and the carotid in some instances has had to be tied. I once had a serious hemorrhage to deal with some twenty-five years ago. The usual styptics, and even the cautery, failed to relieve it. At last I tried a remedy, which I have used ever since with perfect success. A chemist had informed me, a short time before, that a small quantity of gallic acid would prevent tannic acid dissolving. I mixed two parts of the tannic and one of the gallic in a little water, and gave the patient two teaspoonfuls, telling him to sip them slowly. The bleeding stopped almost at once. We have since used the preparation at the Throat Hospital, and always with perfect success. The patient must be told to *swallow* the liquid, not gargle. Application with a brush will do no good. He should swallow the fluid slowly, as if it were difficult to get it down, and must on no account wash out his mouth or gargle.—*Edinburgh Medical Journal.*

**Lanolin.**—In a recent number of the *Therapeutische Monatshefte*, Dr. A. Gottstein, of Berlin, writing of sublimated lanolin as an antiseptic, refers to a former communication of his maintaining that lanolin is proof against destruction by micro-organisms, and that a layer of it, even without the addition of any antiseptic, resists the passage of germs. As a vehicle for antiseptics, it is therefore far superior to glycerin and to other fats.—*Ex.*

**Progress in Otology.**—In a paper read before Ky. State Medical Society, Dr. S. G. Dabney says: The most valuable of all agents for cleansing the ear is peroxide of hydrogen. Since cleanliness is the prerequisite to the treatment of pus formations in the ear, this agent becomes invaluable. In a paper commending its value, read before the Louisville Medico-Chirurgical Society, I drew the following conclusions from its use in ear diseases:

1. Owing to the strong power possessed by the peroxide of hydrogen as a pus-destroying agent, it is invaluable in the treatment of purulent accumulations, especially when situated in cavities where ordinary methods for their removal are insufficient.
2. In acute purulent inflammation of the middle ear it will, unassisted, cut short the discharge in from three to ten days.
3. In chronic suppuration of the middle ear unaccompanied by necrosis, as a preliminary cleaning agent to the boric-acid treatment, it aids materially the therapeutic powers of this method.
4. It hastens rapidly the healing of abscesses connected with or due to necrosed bone, as shown by its use in mastoid disease.

Politzer, in 1880, recommended pilocarpine in recent cases of labyrinthine disease, on the theory that it stimulated the secretions, and thus hastened the absorption of the exudate. Kostegarten, on the same principle, tried it in old-standing diseases of the middle ear. He says it causes an exudation, and from this ensues pliabil.ty of the sclerosed tissues, moistening and softening adhesions, and thus the conducting apparatus becomes more movable and capable of transmitting sohund vibrations.

While great advancements have been made in the therapeutics of chronic suppuration of the middle ear, there remains a certain number of these cases that resist all medicinal application. Dr. Sexton, of New York, now recommends for these cases surgical methods of treatment. In general surgery it is familiar to all that chronic suppuration is always due to obstructed drainage or to disease of the deep tissues, usually bone. So, in chronic ear discharges there is obstruction to a free outflow of the pus, due to a circumscribed necrosis of the fundus of the ear. The rational treatment here is to give exit to the pus, and cleanse the cavity with antiseptic solutions.

**Uralium: A new Hypnotic.**—Gustavo Poppi, a medical student of Bologna, recently described to the Medico-Chirurgical Society of that city the effects of a new hypnotic produced by the combination of chloral hydrate with urethan. From experiments on animals and on the human subject he concludes that this substance—*uralium*—induces sleep more quickly and more certainly than any other known hypnotic. It causes no bad effects of any kind. It has been given in cases of heart disease and nervous complaints with the best results, even when other hypnotics had failed. The *British Medical Journal* says that experienced practitioners will recognize in Signor Poppi's enthusiastic account of his discovery the familiar trumpet-blast that heralds the first appearance of so many new remedies, "which have their day and cease to be," or which, at any rate, soon lose their title to therapeutic infallibility.—*Chemist and Druggist.*

**How to clean Hypodermic Syringes.**—Syringes, whose canals have become obstructed so that a fine wire cannot be drawn through, are cleaned by holding them for a moment over a flame; the foreign substance is thus quickly destroyed and driven off. If a wire has been rusted into the needle, it should be dipped in oil before holding over the flame. To remove the rust from the interior of the canula, it is well to pass oil through the canula, then heating it; then rinse it out with alcohol. The needle is then ready for use.—*Deutsch Med. Wochenschr.*, 1889.

